# Indicators of guideline-concordant care in lung cancer defined with a modified Delphi method and piloted in a cohort of over 5,800 cases

**DOI:** 10.1186/s13690-021-00528-0

**Published:** 2021-01-25

**Authors:** Anita Andreano, Maria Grazia Valsecchi, Antonio Giampiero Russo, Salvatore Siena, on behalf of the Lombardy Lung Cancer Working Group, Milena Andruccioli, Antonio Ardizzoia, Agnese Assi, Alessandro Baisi, Amedeo Vittorio Bedini, Giordano Beretta, Maurizio Bersani, Francesco Bertagna, Elisabetta Berti, Alessandro Bertolini, Anna Bettini, Mario Bignardi, Francesco Bini, Claudio Bnà, Francesca Bono, Daniele Bonora Ottoni, Andrea Borghesi, Paolo Borghetti, Antonio Brunelli, Alberto Buffoli, Carlo Cacioppo, Angelomaria Calati, Anna Calcagno, Sabrina Casagrande, Clelia Casartelli, Massimo Castellani, Antonino Castro, Gianpiero Catalano, Laura Catena, Giovanni Luca Ceresoli, Massimiliano Cergnul, Gianluigi Ciocia, Annamaria Colombo, Barbara Conti, Daniela Corti, Diego Cortinovis, Claudio Cozzi, Flavio Crippa, Andrea De Monte, Daniela De Candis, Domenico De Toma, Filippo De Marinis, Biancamaria DiMarco, Franca Di Nuovo, Claudio Doglioni, Valentina Fabbris, Paolo Faccioli, Daniele Fagnani, Gabriella Farina, Laura Fariselli, Virginio Filipazzi, Francesca Franzi, Paolo Frata, Ana Maria Samanes Gajate, Gianstefano Gardani, Massimo Gasparini, Monica Giordano, Fabrizio Grignani, Claudio Landoni, Carlo Lombardi, Fabrizio Lombardi, Andrea Luciani, Lorenzo Stefano Maffioli, Roberta Marchione, Paola Mariani, Anna Maspero, Fausto Meriggi, Aurora Miedico, Patrizia Morbini, Giovanni Muriana, Giulio Orlandoni, Enrica Paris, Paolo Pedrazzoli, Giuseppe Pepe, Ezio Pezzica, Fabio Piccoli, Gianpaolo Pinotti, Antonello Quadri, Andrea Ravasio, Franco Ravenna, Elisabetta Ricchiuti, Davide Rinaldo, Gianni Roda, Gerolamo Rossi, Armando Santoro, Carlo Pietro Soatti, Francesco Stiglich, Luca Tagliabue, Valeria Tirelli, Andrea Tironi, Maurizio Tomirotti, Laura Lavinia Travaini, Giovanni Ucci, Adriano Vaghi, Angelo Vanzulli, Giulia Veronesi, Isabella Vittimberga, Claudia Zambelli

**Affiliations:** 1 Epidemiology Unit, Agency for Health Protection of Milan, C.so Italia 19 –, 20122 Milan, Italy; 2 Center of Biostatistic for Clinical Epidemiology, School of Medicine and Surgery, University of Milan Bicocca, Monza, Italy; 3 Niguarda Cancer Center, Grande Ospedale Metropolitano Niguarda and Department of Oncology and Hemato-Oncology, Università degli Studi di Milano, Milan, Italy

**Keywords:** Lung neoplasms, Quality indicators, health care, Guideline adherence, Quality of health care, Health information systems

## Abstract

**Background:**

To identify indicators of guideline-concordant care in lung cancer, to implement such indicators with cancer registry data linked to health databases, and to pilot them in a cohort of patients from the cancer registry of the Milan Province.

**Methods:**

Thirty-four indicators were selected by revision of main guidelines by cancer epidemiologists, and then evaluated by a multidisciplinary panel of clinicians involved in lung cancer care and working on the pathway of lung cancer diagnosis and treatment in the Lombardy region, Italy. With a modified Delphi method, they assessed for each indicator the content validity as a quality measure of the care pathway, the degree of modifiability from the health professional, and the relevance to the health professional. Feasibility was assessed using the cancer registry and the routine health records of the Lombardy region. Feasible indicators were then calculated in the cohort of lung cancer patients diagnosed in 2007–2012 derived from the cancer registry of the Milan Province. Criterion validity was assessed reviewing clinical records of a random sample of 114 patients (threshold for acceptable discordance ≤20%). Finally, reliability was evaluated at the provider level.

**Results:**

Initially, 34 indicators were proposed for evaluation in the first Delphi round. Of the finally 22 selected indicators, 3 were not feasible because the required information was actually not available. The remaining 19 were calculated on the pilot cohort. After assessment of criterion validity (3 eliminated), 16 indicators were retained in the final set and evaluated for reliability.

**Conclusion:**

The developed and piloted set of indicators is now available to implement and monitor, over time, quality initiatives for lung cancer care in the studied health system.

**Supplementary Information:**

The online version contains supplementary material available at 10.1186/s13690-021-00528-0.

## Background

Lung cancer remains a killer disease being the leading cause of cancer death for both genders in countries with a high Human Development Index (HDI), with world age-standardized mortality rates of 36.4 per 100,000 for males and 14.6 per 100,000 for females, accounting for approximately 22% of all cancer deaths in 2018 [1]. This despite the discontinuation in smoking habit occurred in high HDI countries that led to a reduction of incidence rates [2].

There is accumulating evidence that patients not receiving guideline concordant treatments have worse outcomes [3, 4]. Hence, it is important to define and implement a set of indicators to verify concordance between guidelines and care delivered in clinical practice. Previous studies defining indicators to evaluate guideline-concordance in lung cancer care have been carried out in The Netherlands [5, 6] and Ontario [7]. However, to serve the scope of quality of care improvement, the whole process from definition, to operationalization and pilot testing needs to be carried out in a specific health service [8, 9]. Also, indicators have to be timely, reliable and economically computable on a large scale to inform quality improvement activities [10, 11]. In the oncologic setting, this is impractical if indicators are retrospectively computed using data abstracted ad hoc from clinical records, while it is expected to be feasible using current health database linked to modern population cancer registers [12–14].

The aim of this project was to develop, with a multidisciplinary panel, a set of adherence to guidelines indicators concerning aspect of diagnosis, treatment and follow-up of lung cancer care and to pilot them in a cohort of lung cancer patients from the Cancer Registry of the Milan Province, a territory covered by the Agency for Health Protection of Milan, Italy.

## Methods

### Identification and selection of the indicators

We first identified relevant evidence-based practice guidelines and potential indicators from the literature and then, with a modified Delphi method, used clinical experts’ knowledge to select the final set of indicators in two rounds. The whole process is summarized in Supplementary Figure 1. On 07 May 2015 we interrogated the SAGE clinical guidelines database [15] (Supplementary Material), that collects and evaluates according to the scheme AGREE II all published guidelines in English [16]. From the retrieved list, we selected guidelines with a score ≥ 50% for the ‘rigour’ and ≥ 30% for the ‘applicability’ domain. Additionally, we used the 2013 Lung Cancer Guidelines of the Italian Association of Medical Oncology (AIOM), which are published in Italian language [17]. Those guidelines are developed according to the Scottish Intercollegiate Guidelines Network (SIGN) and Grading of Recommendations Assessment, Development and Evaluation (GRADE) methodologies [18, 19]. Systematic evaluation and selection of recommendations from the identified guidelines was carried out by one of the authors, and reviewed by a senior author. We included all recommendations whose compliance could be evaluated by process or short term outcomes indicators, and that could be theoretically calculated with data available from the cancer register and current health databases of the Lombardy Region. We excluded all recommendations that required data unavailable in the health databases to be verified (e.g. results of patient performance status assessment, advices given to patients). To identify previously developed indicators for lung cancer care evaluation, we searched the Pubmed database with no language and date restrictions on 20 April 2015 (Supplementary Material – Search strategy). We also searched the National Quality Measures Clearinghouse database on 05 May 2015 [20]. After screening on title and abstract, full-text of the relevant publications were examined. One of the author extracted all the indicators and matched them with one of the selected recommendation. If more than one indicator matched with a recommendation, we combined them into a single indicator. If there were minor differences in time windows or definition of the denominator, we maintained the more suitable for the database and health system under scrutiny. If a recommendation had no corresponding published indicator, a new one was proposed. This process was reviewed by other authors. The provisional list of indicators was then specified unambiguously and exhaustively in term of numerator, denominator, and data sources, and then organized in a questionnaire using the freely available survey tool eSurv (https://esurv.org/). The survey consisted of five sections evaluating the following dimensions: organization, diagnosis, surgery, medical treatment and follow-up. Each indicator was presented with a title and a description of how the indicator would have been calculated from the register and the administrative data. Below it was asked to rate, using a seven-point Likert scale, the validity of the indicator as a quality of care measure, the possibility to modify the value of the indicator at the patient level by the health professional, and the usefulness of the indicator for self-assessment. Definitions provided to the panel are provided in English translation in the Supplementary Material. A free text field allowed to suggest changes to the indicator wording or to propose an alternative indicator. Panel designation is described in Supplementary Material. Participants were chosen on agreement to participate basis. Results of the first round were evaluated, both in terms of obtained scores and comments expressed in the free-text field. An indicator was retained if at least 75% of the panel members completing the questionnaire scored the validity question equal or greater than five, and at least 50% of them scored the modifiability and utility question equal or greater than four. The reduced list of indicators was also updated according to relevant comments in the free-text field. A summary of the results of the first round was sent to the clinicians who completed the questionnaire, including rates and proposed modifications. The second round was conducted during an in-person meeting, run as an informal discussion, with representatives of the panel.

### Identification of the cohort and pilot calculation of the indicators

We calculated all the finally selected indicators on a pilot cohort derived from Cancer Registry of the Agency for Health Protection of Milan, Italy including all patients developing a lung cancer (International Classification of Diseases for Oncology version 3, first revision (ICDO-3) topographic codes C33–34) in the period 2007–2012 and registered with the Lombardy Regional Health Service. A subset of the area covered by the Agency was used, corresponding to fourteen municipalities around Milan with a total population of 1.546.237 inhabitants at 01/01/2013 [21]. The cancer register is accredited at the national level and included in the Volume XI of Cancer Incidence in Five Continents [22]. It is semi-automated, using multiple sources of information (i.e. inpatient, histopathology and death certificate databases) and a record linkage algorithm to match all information at the individual level [23] with the review of all cases for the assignment of morphology and stage. We calculated a modified version of the Charlson comorbidity index using both in and outpatient databases [24, 25]. The date of incidence was defined, according to international cancer registration rules [26]. Exclusion criteria were: tumors identified only through death certificate and mesenchymal histology. For indicators applying to either small cell (SCLC) or non-small cell lung cancer (NSCLC), patients were classified as SCLC if the histology ICD-O-3 code was 8041–8045 and NSCLC in all other cases, including nonspecific histology codes and not cyto-histologically confirmed cases. To calculate the indicators at patient level we used the register and all available computerized sources of health information from January 2006 to December 2016, including outpatient diagnostic and therapeutic procedures, inpatient, prescription, and emergency access databases. We derived gender, age and stage at diagnosis from the register. First therapy was defined as described in Supplementary material. For each indicator, we assigned the patient to the hospital where he/she received the relevant procedure for the first time. We assessed feasibility of each indicator verifying that the data needed to calculate the indicators were actually available for the whole population in the administrative data. We assessed construct validity in a stratified (main histological type, stage, type of first treatment) random subsample of 114 patients, verifying that the required coding was reported in the administrative databases when the procedure had actually been performed according to the full clinical record, and reported number and percentage of discordances. The threshold of acceptable discordances to retain the indicator was set at equal or lower than 20%. We first computed the indicators overall, and in relevant subgroups of patients, and the assessed their clinimetric properties. Potential improvement was evaluated analyzing median value. We also computed number of providers scoring either 0 (flooring effect) or 100% (ceiling effect). Finally, we evaluated the reliability of the indicators, that is a measure that can vary between 0 and 1 and describes how well one can confidently distinguish the performance of one provider from another measuring the signal (variability of the indicator that can be attributed to real differences in performance) to noise (random variability of the indicator value) ratio [27]. We calculated the reliability of each indicator for all providers, transformed it to percentage to interpret as the percentage of variability that can be attributed to real difference in performances, and reported the number of providers with a reliability equal or greater than 70%, which is an accepted cut-off for good reliability [28, 29].

### Statistical analysis

Descriptive statistics including quartiles were calculated for the validity, modifiability and utility scores received by each indicator in the first Delphi round. Differences in the distribution of covariates across gender, stage or years were assessed using the Manthel-Haenszel test, analysis of variance or Wilcoxon test, as appropriate. All tests were two-sided with a significance level of 0.05. We calculated each indicator as the proportion of patients who received the procedure in the defined time window among those defined as eligible. Indicators were calculated also stratified by age (≤60, 61–70, ≥70 years), sex, Charlson index (0, 1–2, ≥3) and stage (I to IV). To assess reliability, we measured noise as the variance of a proportion, and signal using the variance of the random effect of a hierarchical logistic model with the indicator value as the outcome, age, gender, stage and Charlson index as first level predictors, and provider as the only second level variable. All analyses were performed with SAS software (v.9.4, SAS Institute, Cary NC).

## Results

### Review of the literature and identification of candidate indicators

The search of the SAGE clinical guidelines database retrieved 45 records (Supplementary Figure 1). Ten guidelines did not include NSCLC or SCLC, 11 had no AGREE II evaluation, and 18 guidelines had a rigour < 50% or an applicability < 30%. The six remaining guidelines were used to extract recommendations [30–35] .The literature search for lung cancer indicators retrieved 557 records. Based on title and/or abstract, *n* = 532 were excluded as not relevant and *n* = 8 because they were guidelines already included in the SAGE database search results. Three manuscripts were excluded on the basis of the full-text evaluation [36–38]. Indicators were extracted from the 14 remaining papers [7, 9, 39–41] and matched with a guidance. A set of *n* = 34 indicators, each assessing a recommendation, was included in the web-based questionnaire for the first round of the Delphi process.

### Results of the Delphi process

A total of 225 physicians were invited to participate to the Delphi process. The number of questionnaires compiled at least partially was 95 (42% of respondents); 85 questionnaires were completed in full. Thirteen indicators did not satisfy the selection criteria and were excluded from the second round. One additional indicator evaluating time from thorax computed tomography (CT) to surgery was proposed by panel members (O4). Four indicators were partially modified based on the comments received in round 1. The meeting constituting the second round led to approval of the changes in the indicator formulation but did not modify the number of indicators. Table 1 reports the finally 22 selected indicators with first round scores.

**Table 1:** Indicators (No. 22) to measure guideline-concordant care in lung cancer patients selected with a modified Delphi process

	Short name	Numerator^a^	Denominator	No.of respondents	Validity	Modifiability	Utility
					M (Iq-IIIq)	M (Iq-IIIq)	M (Iq-IIIq)
O1	First contact to first therapy ≤60 days	With an interval between first contact and first therapy ≤60 days	All patients with any recorded treatment and a contact^§^ ≤ 180 days	95	6 (5–6)	4 (3–5)	6 (5–6)
O2	From PET to surgery ≤45 days	With an interval between PET and surgery ≤45 days	All patients receiving lung surgery and having a PET within 3 months before	95	6 (5–7)	4 (3–5)	5 (4–6)
O3	Multidisciplinary evaluation	With multidisciplinary evaluation within 30 days before first treatment	All patients with any recorded treatment	95	6 (5–7)	5 (4–6)	6 (5–7)
O4^b^	From thorax CT to surgery ≤45 days	With an interval between thorax CT and surgery ≤45 days	All patients receiving lung surgery and having a thorax CT within 3 months before		na	na	na
D1	Thorax CT at diagnosis	Thorax CT within 2 months from diagnosis	All patients	92	6 (5–7)	4 (2–6)	5 (3–6.5)
D2	Thorax CT before biopsy	With a thorax CT in the 30 days preceding broncoscopy or other biopsy procedure	All patients receiving a biopsy within 3 months before and one month after diagnosis	92	6 (5–7)	4.5 (3–6)	6 (4–7)
D3	Treatment with curative intent preceded by PET	With a PET record in the 3 months preceding surgery or chemoradiation	NSCLC patients in stage I-III receiving either surgery or concomitant/sequential chemo-radiation	92	6 (5–7)	4 (3–6)	6 (4–7)
D4	Cyto-histologic confirmation	With cyto-histologic confirmation in the cancer register	All patients	92	6.5 (5–7)	4 (3–6)	6 (5–7)
D5	NSCLC Stage III patients assessed for metastasis before curative intent treatment	With a head CT/MR and PET/bone scan in the month preceding first therapy	NSCLC in stage III and receiving concomitant/sequential chemo-radiation	92	6 (5–7)	5 (3–6)	6 (4–7)
D6	SCLC patients fully staged	With a thorax CT and abdominal CT/sonography and head CT/MR and PET/bone scan in the 3 months before diagnosis	All SCLC patients	92	6 (5–7)	5 (3–6)	6 (5–7)
S1	Survival after first surgery	Not deaceased within 30 days from first surgery	All patients receiving lung surgery	90	6 (6–7)	4.5 (2–6)	6 (5–7)
S2	Patients with a thorax CT ≤30 days before surgery	thorax CT in the 30 days before first surgery	All patients receiving lung surgery	90	6 (5–7)	4 (3–6)	6 (5–7)
S3	Functional evaluation before surgery	Lung functionality evaluation in the month before first surgery	All patients receiving lung surgery in stage I-IIIa	90	7 (6–7)	5 (3–6)	6 (5–7)
S4	Stage I-IIIA NSCLC patients undergoing curative intent surgery	Receiving surgery with presumed curative intent	NSCLC patients in stage I-IIIa	90	6 (5–7)	5 (3–6)	6 (5–7)
S5	Stage I-IIA NSCLC patients undergoing lobectomy	Receiving lobectomy as first surgery	NSCLC patients in stage I-IIa and receiving lung surgery	90	6 (5–7)	4 (3–6)	6 (5–6)
S6	No second surgery within 30 days	Not undergoing a second lung intervention within 30 days	All patients receiving lung surgery	90	6 (6–7)	4 (2–6)	6 (5–7)
S7	Hospital stay ≤14 days for first surgery	With an hospital stay ≤14 days and with no hospital access in the 30 days after discharge	All patients undergoing segmentectomy, lobectomy or pneumonectomy as their first lung surgery	90	6 (5–7)	4 (3–6)	6 (5–7)
M1	Stage II-III NSCLC patients receiving chemo-radiation	Receiving concomitant or sequential chemo-radiation	NSCLC patients in stage II-III that did not receive surgery	87	5 (5–6)	4 (3–5)	5 (4–6)
M2	SCLC patients undergoing medical oncologic therapy or radiotherapy	Receiving medical oncologic treatment and/or radiotherapy	SCLC patients not in stage IV	87	6 (5–7)	4 (3–5)	5 (4–6)
M3	Palliative care before death	Home-care, hospice or hospital admission for palliative care in the 3 months before death	All patients deceased at 31/12/2016	85	6 (6–7)	5 (3–6)	6 (5–7)
M4	Pain management before death	With an opioids prescription in the 3 months before death	All patients deceased at 31/12/2016	85	6 (5–7)	5 (3–6)	6 (4–7)
F1	Follow-up in year 2, 3, and 4 for surviving patients	With at least one follow-up visit or hospital admission in the year (excluding urgent admission and admission for medical oncologic treatment, radiotherapy or lung surgery)	Patients alive after 2, 3, and 4 years	85	6 (5–7)	5 (3–6)	6 (5–7)

*Abbreviations*: *M* median, *I q* First quartile, *III q* Third quartile, *CT* Computed-tomography, *PET* Positron emission tomography

^a^for all indicators, patients included in the dominator and having the characteristic described in the ‘numerator’ column

^b^indicator proposed by the panel a b in the first round

### Pilot cohort

In the 2007–2012 period there were 5860 cases of lung cancer (International Statistical Classification of Diseases and Related Health Problems 10th Revision (ICD10) topographic codes C33–34) in the resident population of the Milan province. Death certificate only (*n* = 88) and mesenchymal histology (*n* = 26) cases were excluded. This left *n* = 5746 patients, 91% NSCLC and 9% SCLC (Supplementary figure 2). Seventy-five percent were males and 55% of cases were in the 66–80 years range (Table 2). A significant increase in the proportion of female was observed over time from 24% in 2007 to 28% in 2012 (*p* = 0.009), while there were no significant changes in age distribution (*p* = 0.8). Overall 43% of patients had a Charlson comorbidity index greater than zero, with males having more comorbidities (mean Charlson index 1.5 for male vs. 1.2 for females, *p* < 0.001). No differences in Charlson index distribution were observed with stage (*p* = 0.5).

**Table 2:** Lung cancer patient cohort in the Milan province, Italy (*n* = 5746 subjects)

	No.	(%)
Year of incidence		
2007	909	(15.8)
2008	952	(16.6)
2009	1015	(17.7)
2010	975	(17.0)
2011	945	(16.5)
2012	950	(16.5)
Gender		
Male	4310	(75.0)
Female	1436	(25.0)
Age class (years)		
≤ 55	422	(7.4)
56–60	496	(8.6)
61–65	797	(13.9)
66–70	1010	(17.6)
71–75	1163	(20.2)
76–80	1002	(17.4)
> 80	856	(14.9)
Charlson index		
0	3256	(56.7)
1	1226	(21.3)
2	627	(10.9)
≥ 3	334	(5.8)
Stage		
No. missing	879	(15.3)
I	611	(12.6)
II	526	(10.8)
III	1068	(21.9)
IV	2662	(54.7)
Cito-pathologically confirmed		
No	932	(16.2)
Yes	4814	(83.8)
Histology groups^a^		
NSCLC	4304	(89.4)
SCC	975	(20.3)
adenocarcinoma	2194	(45.6)
large cell	151	(3.1)
other, specified	810	(16.8)
aspecific	174	(3.6)
SCLC	510	(10.6)
Treatment		
No recorded treatment	2350	(40.9)
Surgery	1124	(19.6)
Chemo-radiotion	388	(6.8)
Chemotherapy	1570	(27.3)
Radiotherapy	314	(5.5)

*Abbreviations*: *NSCLC* Non-small-cell lung carcinoma, *SCC* Squamous cell carcinoma, *SCLC* Small-cell lung carcinoma

^a^in microscopically confirmed cases

### Clinimetric properties of the indicators

Indicators were calculated on the pilot cohort and their values are reported in Table 3. Regarding feasibility, the indicator ‘palliative care before death’ (M3) was actually not feasible because the required administrative datasets were not available for the entire population, i.e. available for some municipalities but not others. The same was true for ‘Multidisciplinary evaluation’ (O3), as the code was used only from 2 out of 92 providers in the analyzed years, and ‘Functional evaluation before surgery’ (S3) as the required exams were systematically never billed in the pre-admission consultation to surgery.

**Table 3:** Value of the indicators to measure guideline-concordant care in lung cancer patients of the 2007–12 pilot cohort of the Milan province (*n* = 5746 subjects), overall and in subgroups of patients

	Indicator	Overall			Age class			Gender		Charlson index			Stage			
		No.	Total No.	%	≤60	61–70	≥70	Male	Female	0	1–2	≥3	I	II	III	IV
O1	First contact to first therapy ≤60 days	1750	2663	65.7	72.1	66.4	61.8	65.2	67.4	67.9	64.0	54.5	49.1	63.9	65.1	74.3
O2	From PET to surgery ≤45 days	518	706	73.4	85.0	74.3	67.1	73.9	72.0	76.0	69.7	68.4	69.9	78.4	70.4	75.4
O4	From thorax CT to surgery ≤45 days	491	759	64.7	74.5	64.6	60.5	65.5	62.6	65.5	66.4	54.2	59.6	69.2	67.3	67.5
D1	Thorax CT at diagnosis	4416	5746	76.9	78.2	75.8	77.1	77.1	76.1	73.9	79.6	83.8	71.4	72.6	78.7	82.9
D2	Thorax CT before biopsy	2487	3283	75.8	78.9	74.5	75.5	75.7	76.0	74.9	77.2	75.8	60.1	68.5	75.4	83.6
D3	Treatment with curative intent preceded by PET	1098	2125	51.7	48.4	42.4	57.1	51.6	51.9	48.1	52.9	62.1	42.1	40.3	57.6	
D4	Cyto-histologic confirmation	4814	5746	83.8	94.1	90.5	76.6	84.3	82.2	88.1	79.7	73.5	92.6	91.6	86.6	80.5
D5	NSCLC Stage III patients assessed for metastasis before curative intent treatment	166	403	41.2	48.8	40.9	37.9	42.5	37.3	43.0	39.3	34.6			41.2	
D6	SCLC patients fully staged	50	510	9.8	10.0	14.0	6.6	8.8	12.9	12.0	6.2	8.8	16.7	0.0	12.6	8.0
S1	No death after first surgery	9	1124	99.2	100	98.9	99.2	98.9	100	99.7	98.9	96.8	100	98.7	99.4	97.1
S2	Patients with a thorax CT ≤30 days before surgery	330	1124	29.4	34.7	31.2	25.5	30.2	27.1	28.0	31.2	31.6	24.5	33.3	28.0	39.4
S4	Stage I-IIIA NSCLC patients undergoing surgery with curative intent	897	1768	50.7	72.9	59.6	23.7	50.1	52.4	60.5	60.0	42.1	55.4	48.3	35.8	
S5	Stage I-IIA NSCLC patients undergoing lobectomy	687	1005	68.4	72.5	68.8	66.4	66.6	73.2	71.2	65.0	61.4	67.7	75.1	68.2	
S6	No second surgery within 30 days	1117	1124	99.4	99.0	99.1	99.8	99.4	99.3	99.1	99.7	100.0	99.1	100.0	99.4	100.0
S7	Hospital stay ≤14 days for first surgery	611	930	65.7	76.5	64.8	61.9	62.3	75.1	68.6	63.3	53.3	71.7	64.6	58.6	53.5
M1	Stage II-III NSCLC patients receiving chemo-radiation	46	977	4.7	9.8	6.1	2.8	4.9	3.9	5.1	5.7	0.8		2.0	5.4	
M2	SCLC patients undergoing medical oncologic therapy or radiotherapy	143	234	61.1	75.0	65.5	53.2	59.5	65.6	65.1	60.3	46.7	50.0	56.3	65.4	
M4	Pain management before death	4414	4798	92.0	92.1	92.2	91.8	92.4	90.7	91.0	92.8	94.3	88.8	91.2	94.1	92.1
F1	Follow-up in year 2 for surviving patients	1122	1552	72.3	69.1	74.5	71.7	72.9	70.8	70.0	76.3	73.7	71.3	70.4	82.2	74.4
	Follow-up in year 3 for surviving patients	861	1169	73.7	74.2	74.7	72.4	73.9	73.2	72.0	76.9	73.8	75.3	72.4	80.9	66.1
	Follow-up in year 4 for surviving patients	715	892	80.2	77.5	83.2	78.2	80.2	80.0	78.8	83.3	78.9	83.5	79.6	82.3	65.0

Three of the indicators did not meet the criterion validity threshold (i.e. 20% of discordances) and were eliminated (Table 4): ‘Treatment with curative intent preceded by PET’ (D3), ‘SCLC patients fully staged’ (D6), and ‘Patients with a thorax CT ≤30 days before surgery’ (S2). The indicator ‘SCLC patients undergoing medical oncologic therapy or radiotherapy’ (M2) did not have a high reliability (≥ 70%) in none of the 56 evaluated providers (Table 5). Also, the indicators O1, O4, D1 and S5 had a high reliability in less than 50% of the evaluated providers.

**Table 4:** Summary of the evaluation of construct validity, through clinical records evaluation, of a set of indicators to measure guideline-concordant care in lung cancer patients in a subsample of 114 patients from a pilot population of 5746 subjects

	Indicator	No. discordant^**a**^	No. evaluated	% discordant^**a**^
O1	First contact to first therapy ≤60 days	17	97	17.5
O2	From PET to surgery ≤45 days	1	16	6.3
O4	From thorax CT to surgery ≤45 days	1	16	6.3
D1	Thorax CT at diagnosis	12	114	10.5
D2	Thorax CT before biopsy	15	79	19.0
D3	Treatment with curative intent preceded by PET	10	38	**26.3**^b^
D4	Cyto-histologic confirmation	0	114	0.0
D5	NSCLC Stage III patients assessed for metastasis before curative intent treatment	4	22	18.2
D6	SCLC patients fully staged	18	28	**64.3**^b^
S1	No death after first surgery	0	25	0.0
S2	Patients with a thorax CT ≤30 days before surgery	6	25	**24.0**^b^
S4	Stage I-IIIA NSCLC patients undergoing surgery with curative intent	0	56	0.0
S5	Stage I-IIA NSCLC patients undergoing lobectomy	0	24	0.0
S6	No second surgery within 30 days	0	25	0.0
S7	Hospital stay ≤14 days for first surgery	0	20	0.0
M1	Stage II-III NSCLC patients receiving chemo-radiation	0	40	0.0
M2	SCLC patients undergoing medical oncologic therapy or radiotherapy	0	21	0.0
M4	Pain management before death	1	90	1.1

The indicators Follow-up in year 2, 3 and 4 (F1) for surviving patients were not evaluated as full clinical records, including outpatient visit reports, were not available for the follow-up period

^a^procedure performed and in the correct time frame according to clinical record (indicator = 1) and corresponding codes not found in administrative databases in the correct time frame (indicator = 0). ^b^ indicator with discordance percentage higher than 20%, that was set as the a-priori acceptable threshold value

**Table 5:** Summary of the evaluation of reliability, ceiling effect assessment and distribution across providers of a set of indicators to measure guideline-concordant care in lung cancer patients in a pilot population of 5746 subjects

	Indicator	No. of patients	No. of providers with			Providers with reliability ≥ 70%		Value of the indicator across providers				
			≥ 1 evaluated patient	indicator value of 0%	indicator value of 100%	No.	%	Mean	SD	Median	I quartile	III quartile
O1	First contact to first therapy ≤60 days	2386	58	2	10	12	21	69	21	68	57	80
O2	From PET to surgery ≤45 days	706	37	1	15	22	59	79	24	85	64	100
O4	From thorax CT to surgery ≤45 days	759	37	0	10	12	32	72	21	70	54	100
D1	Thorax CT at diagnosis	3086	64	5	17	26	41	78	26	81	73	100
D2	Thorax CT before biopsy	2779	76	4	22	47	62	74	27	79	57	100
D4	Cyto-histologic confirmation	3086	64	4	20	46	72	86	25	97	81	100
D5	NSCLC Stage III patients assessed for metastasis before curative intent treatment	353	37	11	5	19	51	34	34	29	0	52
S1	No death after first surgery	1124	39	1	34	37	95	96	16	100	100	100
S4	Stage I-IIIA NSCLC patients undergoing curative intent surgery	1216	47	9	11	39	83	59	38	71	18	94
S5	Stage I-IIA NSCLC patients undergoing lobectomy	1005	39	5	6	11	28	61	30	63	50	80
S6	No second surgery within 30 days	1124	39	0	33	33	85	99	4	100	100	100
S7	Hospital stay ≤14 days for first surgery	930	39	6	7	24	62	59	34	67	26	86
M1	Stage II-III NSCLC patients receiving chemo-radiation	799	92	67	4	71	77	9	22	0	0	5
M2	SCLC patients undergoing medical oncologic therapy or radiotherapy	175	56	12	18	0	0	60	38	71	29	100
M4	Pain management before death	2347	62	3	29	36	58	89	23	98	91	100
F1	Follow-up in year 2 for surviving patients	1167	45	4	8	12	27	71	27	75	65	87
	Follow-up in year 3 for surviving patients	914	43	4	10	15	35	72	28	76	67	92
	Follow-up in year 4 for surviving patients	711	41	4	14	19	46	77	30	89	71	100

*Abbreviations*: *SD* Standard deviation

Calculation of the organizational indicators showed that the proportion of patients having rapid access to treatment after first contact or diagnostic procedures did not differ between men and women and was higher in younger, healthier and more advanced stage patients (O1-O4, Table 3). Half of the hospitals had values of the indicator ‘First contact to first therapy ≤60 days’ (O1, Table 4) equal or lower that 68%. Only 4% of stage II-III NSCLC patients received chemoradiation (M1). ‘Pain management before death’ (M4) had an overall value of 92% but a low improvement potential (median value across providers 98%). Overall, 72% of patients surviving to the end of the second year had a follow-up contact (F1).

## Discussion

This article describes the development, based on the evidence based guidelines and expert consensus, of a set of indicators aimed at monitoring concordance of lung cancer therapy to guidelines and its piloting on 5746 lung cancer patients diagnosed over a 6-year period.

One of the major strengths of this work is the participation of a high number of clinicians involved in the different steps of lung cancer care, guaranteeing that the identified reference guidelines were endorsed and perceived as relevant. Secondly, identifying the cohort with a cancer register allows to adequately define the denominators of the indicators, solving the problem of accurately identify the target population that has been encountered using administrative records only [42]. Also, lung cancer guidelines differ by main histological type (SCLC vs. NSCLC) and by tumor stage. The definition of the cohort from the cancer register also permits to reliably identify the histology.

Lung tumors still have a poor overall prognosis, which is certainly related to the often advanced stage at diagnosis [43]. However, there is evidence that care not concordant with evidence-based guidelines worsen survival and that actual delivered care is often not adherent to those guidelines, especially for advanced stages and SCLC [3, 4, 6, 44, 45]. The report from Nadpara et al., examining a large cohort of lung cancer cases from the Surveillance, Epidemiology, and End Results register linked with Medicare database, investigated variations in guideline-concordant care among the elderly, highlighting that a lower income, increasing age and being non-white reduced the probability to receive guideline-concordant care [4]. The results of our study also highlight differences in adherence to standards with age: older patients received curative surgery less frequently and this was independent from the comorbidity burden.

With the exception of S1 and S6, which are short term outcome-indicators, it was decided to focus on process indicators measuring adherence to guidelines. Process indicators have been criticized as they do not directly measure the outcome [46, 47], which is the main interest both in a patient-centered and public health perspective. A small set of outcome indicators has been developed also for lung cancer [48]. However, process indicators have the advantage to be more sensitive to quality of care differences, not heavily depending on external factors which are not directly under the control of care providers as it happens for outcome indicators [46]. Especially in a setting such not very early lung cancer, where prognosis is still dismal even when receiving the best standard of care, we think that process indicators are fundamental to assess heterogeneity between providers and implement quality improvement actions [49]. Of note, some of the indicators had a low reliability in a large percentage of the providers, suggesting caution in the interpretation of the value of indicators for those providers.

The use of the current administrative databases offers the possibility to implement the indicator on a population scale and to repeat the measurements in time at a reduced cost. However, it introduces some limitations. The first one is that we were not able to calculate three of the indicators produced by the Delphi consensus process because the investigated procedures were not coded in the necessary databases. We also had to drop three indicators having more than 20% discordances between reviewed clinical records and indicators calculated from the administrative databases. However, this is an important information to plan quality improvement initiatives for the future, raising the problem to consistently employ the codes with the clinicians involved in lung cancer care. Also, when using administrative data to calculate health indicators and define case-mix variables (e.g. comorbidity index), concerns about data quality are always present [25, 50, 51], particularly with regard to lack of accuracy in coding and coding variability across professionals. However, they are the larger, more systematic and continuous in time source of health information. Also, specifically to our study, Lombardy health databases are quality checked for reimbursement purposes and have also been found of good quality in several studies [52, 53]. It is also important to acknowledge that not all case-mix variables that are important to fairly compare providers [48] were available such as smoking status and performance status. Finally, the second round of Delphi method was an informal face to face criterion process, that could have been biased by strong opinion leaders. However, after the first round, a high agreement rate was obtained and minor changes were made on the basis of the second round.

## Conclusions

The developed and piloted set of indicators is now available to implement and monitor, over time, quality initiatives for lung cancer care in the studied area.

## Supplementary Information

**Additional file 1: Supplementary methods.** **Supplementary Figure 1.** Process from literature review to indicator calculation and evaluation. **Supplementary Figure 2.** Selection of the pilot cohort of lung cancer patients from the Cancer Register of the Province of XX.

## Data Availability

The dataset from this study is held securely at the ATS of Milan, Epidemiology Unit. Data sharing agreements prohibit the ATS of Milan from making the dataset publicly available. The full dataset creation plan and underlying analytic code are available from the authors upon request.

## Abbreviations

AIOM: Italian Association of Medical Oncology
CT: Computed tomography
GRADE: Grading of Recommendations Assessment, Development and Evaluation
HDI: Human Development Index
ICD10: International Statistical Classification of Diseases and Related Health Problems 10th Revision
ICDO-3: International Classification of Diseases for Oncology version 3
NSCLC: Non-small cell lung cancer
PET: Positron Emission Tomography
SCLC: Small cell lung cancer
SIGN: Scottish Intercollegiate Guidelines Network
