# Supplementary material for: Indicators of guideline-concordant care in lung cancer defined with a modified Delphi method and piloted in a cohort of over 5,800 cases
Source: Arch Public Health. 2021 Jan 25;79:12. doi: 10.1186/s13690-021-00528-0 (PMC7830847; doi:10.1186/s13690-021-00528-0)

**Supplementary material**

**1.Supplementary methods**

**SAGE database search strategy**

The applied search strategy was: “Diagnostic Assessment, Staging, Treatment , Follow Up, Lung, Adult, Radiotherapy, Surgery, Systemic, Biological therapy, Chemotherapy, Hormonal therapy, Guideline is current”. We limited the search to guidelines published after 2010.

**Pubmed search strategy**

("Quality of Health Care"[Mesh:noexp] OR "Quality Indicators, Health Care"[Mesh:noexp] OR "Patient Care Management"[Mesh:noexp] OR "Quality Assurance, Health Care"[Mesh:noexp] OR "Quality Indicator*" OR "Guideline Adherence"[Mesh:noexp] OR "Physician's Practice Patterns/standards"[Mesh]) AND ("Lung Neoplasms"[Mesh] OR (lung[TIAB] AND (cancer[TIAB] OR neoplasm[TIAB] OR tumor[TIAB] OR carcinoma[TIAB] OR malign*[TIAB])))

**Panel designation**

To designate the expert panel, the head of each of the 22 Provincial Departments of Oncology (DIPO) of the Lombardy Region was contacted by email. He/she was asked to select a multidisciplinary group of physicians involved in lung cancer care, without numerical restriction, and to provide their email address. A document describing the project and the instructions to complete the survey were sent to the head of the DIPO two weeks before the opening of the survey, to circulate to the selected DIPO members. An email with the link to the questionnaire was the sent to the physicians, inviting them to complete the questionnaire. A reminder was sent after 7 days.

**Definition given to the panel members to rate indicators using a seven-point Likert scale**

- validity as a quality measure of the diagnostic-therapeutic pathway, or the ability to capture a relevant process in the care pathway of the patient with lung cancer

-possibility of intervention by the operator, or how much of the proposed indicator depends on factors that can be modified directly by the operator (instead of organizational factors such as screening for patients at risk, organization of a pathway between hospital structures, inside the hospital structure or the department)

- usefulness for the operator (doctor or health professional) of the knowledge of the value of the indicator in his own structure, as a self-evaluation of his own work / of the work of the ward or hospital structure

**Definition of first treatment**

We searched all relevant databases for either lung surgery, medical oncologic treatment or radiation therapy in the 3 months preceding and following the incidence date in the register. We assigned the patient to surgery if it was the first therapy or followed a medical oncological treatment within 30 days. If the patient had a medical oncologic treatment as the first therapy and received radiation within four months, or if the first treatment was radiation followed by medical oncologic treatment within four months, it was assigned to concomitant or sequential chemo-radiation.

**2. Supplementary Figures**

**Supplementary Figure 1** Process from literature review to indicator calculation and evaluation


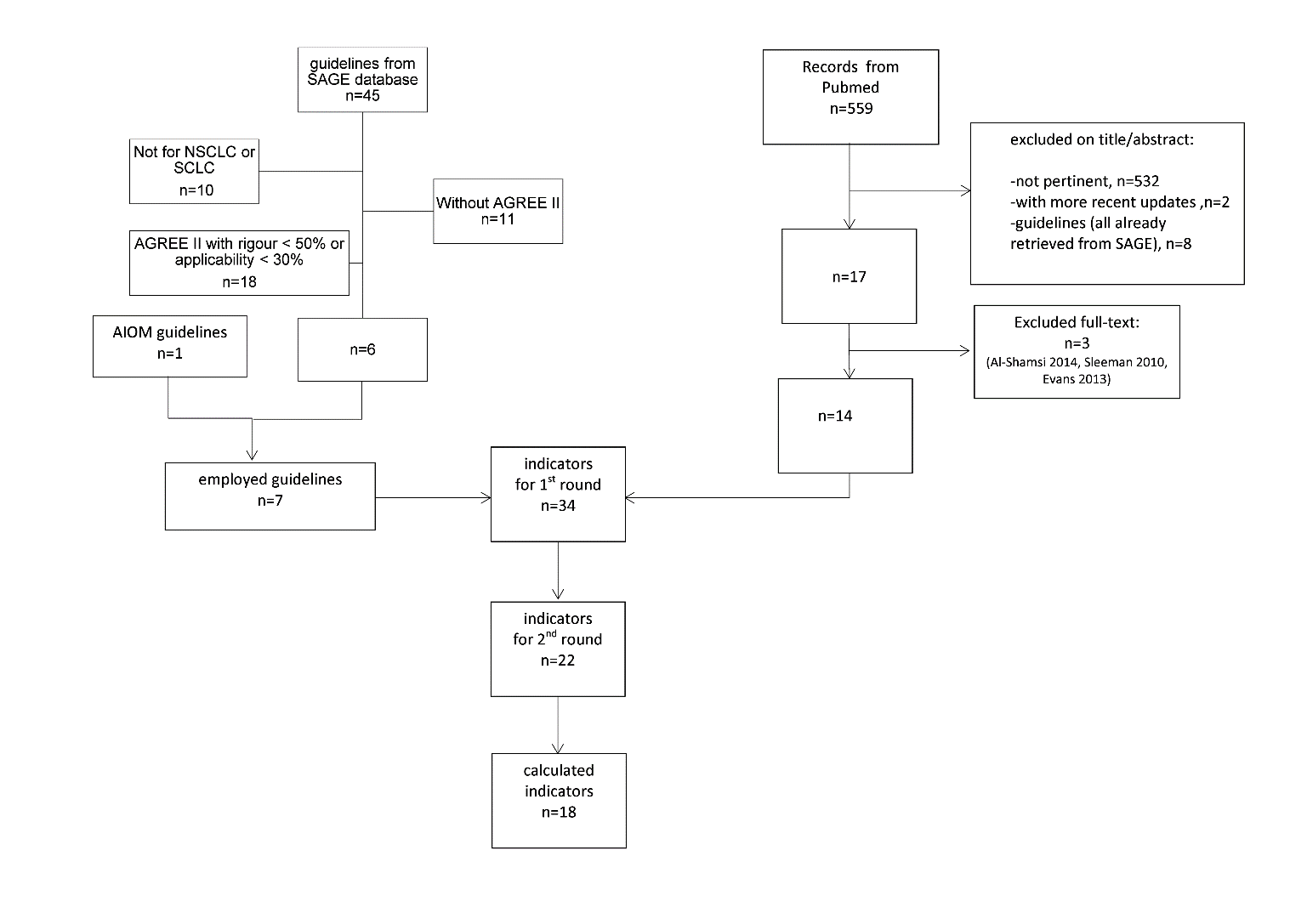


**Supplementary Figure 2** Selection of the pilot cohort of lung cancer patients from the Cancer Register of the Province of XX.


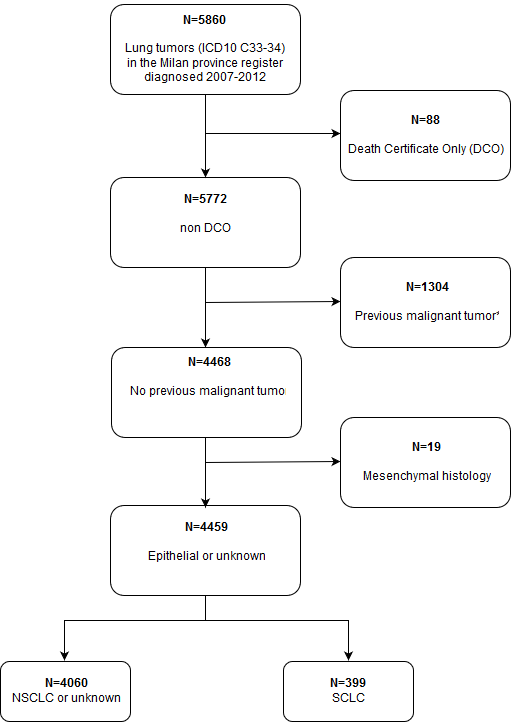

Supplement: Supplementary file 1 — Additional file 1: Supplementary methods. Supplementary Figure 1. Process from literature review to indicator calculation and evaluation. Supplementary Figure 2. Selection of the pilot cohort of lung cancer patients from the Cancer Register of the Province of XX. [file 13690_2021_528_MOESM1_ESM.docx]
